# Oligoglycidol-Functionalised Styrene Macromolecules as Reactive Surfactants in the Emulsion Polymerisation of Styrene: The Impact of Chain Length and Concentration on Particle Size and Colloidal Stability

**DOI:** 10.3390/polym12071557

**Published:** 2020-07-14

**Authors:** Kim Waulthers, Ryan van Zandvoort, Sam Castermans, Jeroen Welzen, Evelien Baeten, Kathleen Stout, Helmut Keul, Daniel Mann, Pascal Buskens

**Affiliations:** 1 Zuyd University of Applied Sciences, Nieuw Eyckholt 300, 6400AN Heerlen, The Netherlands; 1522736waulthers@zuyd.nl (K.W.); 1658905castermans@zuyd.nl (S.C.); jeroen.welzen@chillabs.nl (J.W.); evelien.baeten@zuyd.nl (E.B.); kathleen.stout@zuyd.nl (K.S.); 2 The Netherlands Organisation for Applied Scientific Research (TNO), High Tech Campus 25, 5656AE Eindhoven, The Netherlands; ryan.vanzandvoort@tno.nl; 3 Brightlands Materials Center, Urmonderbaan 22, 6167RD Geleen, The Netherlands; 4 DWI–Leibniz Institute for Interactive Materials, Forckenbeckstrasse 50, 52056 Aachen, Germany; keul@rwth-aachen.de; 5 Institute for Materials Research, Inorganic and Physical Chemistry, Hasselt University, Agoralaan Building D, B-3590 Diepenbeek, Belgium

**Keywords:** polymer nanoparticles, emulsion polymerization, styrene, reactive surfactant, surfmer, oligoglycidol, colloidal stability

## Abstract

Reactive surfactants (surfmers), which are covalently attached to the surface of sub-micron sized polymer particles during emulsion polymerisation, are applied to tailor the surface functionality of polymer particles for an application of choice. We present a systematic study on the use of oligoglycidol-functionalised styrene macromolecules as surfmers in the emulsion polymerization of styrene. Firstly, we report the impact of the surfmer concentration on the particle size for polymerisations performed above and below the critical micelle concentration. Secondly, we report the influence of the oligoglycidol chain length on the particle size. Thirdly, we conducted experiments to analyse the influence of the surfmer concentration and its chain length on the colloidal stability of the aqueous polystyrene nanoparticles in sodium chloride solutions. We demonstrated that the size of polystyrene particles could be influenced by changing both the surfmer concentration and its chain length. Furthermore, we showed that the colloidal stability of the oligoglycidol-functionalized polystyrene particles is dependent on the particle size, and not directly related to the oligoglycidol chain length.

## 1. Introduction

Polymer nanoparticles are widely applied, e.g., in functional nanocomposites [1,2], nano-enabled coatings [3,4], advanced optical materials [5,6] and colloidal molecules [7,8]. For many of these applications controlling the surface functionality of the polymer nanoparticles is essential, e.g., to tune the interfacial tension for successful integration of the particles into composites [2] or coatings [3,4], to add a desired functionality [3,4,9,10], to selectively deposit small metal nanoparticles on the polymer particle surface [11], to create core-shell particles [6,12,13,14] and colloidal molecules [7,15]. The surface functionality of polymer nanoparticles is usually determined by the surfactant used during emulsion polymerisation, which is located on the particle surface. Since regular surfactants are merely attached to the nanoparticle surface via van der Waals forces, they can easily detach from the surface and are also present in the dispersion medium. Reactive surfactants (surfmers) participate in the emulsion polymerisation, and are therefore covalently connected to the particle surface. This facilitates tailoring of the particle surface for the application of choice, and avoids the presence of excess surfactant in the dispersion medium. A range of different surfmers has been reported for the preparation of polymer [8,16,17,18,19,20,21,22,23], inorganic [24,25] and hybrid nanoparticles [26]. One functional moiety that showed great potential in surfmers for the emulsion polymerisation of styrene is oligoglycidol [16]. This functional moiety can be easily modified with a styrene unit for polymerisation [27]. Furthermore, the chain length and number of hydroxyl groups [27] can be varied. It has been shown that oligoglycidol-based surfmers can be used in the emulsion polymerisation of styrene, even below the critical micelle concentration (CMC) [16]. Below the CMC, particle formation is described as homogeneous nucleation, and therefore deviates from micellar nucleation as proposed for Smith–Ewart kinetics [28]. Since relatively high surfmer concentrations are necessary to reach the CMC, the influence of change in nucleation mechanism, from homogeneous to micellar, on the particle size was not analysed yet. Furthermore, the influence of oligoglycidol chain length on the particle size was only partly analysed [16].

Here, we present a systematic study on the influence of the oligoglycidol surfmer concentration and chain length on the polystyrene nanoparticle size in polymerisations with surfmer concentrations above and below the CMC. Furthermore, we analyse the influence of nanoparticle size and oligoglycidol chain length on the colloidal stability in aqueous NaCl solution.

## 2. Materials and Methods

### 2.1. Materials

Ethoxy ethyl glycidyl ether (EEGE) was obtained from 2,3-epoxypropan-1-ol (glycidol) and ethyl vinyl ether according to Fitton et al. [29], purified by distillation and stored under nitrogen atmosphere over molecular sieves (4 Å). Diglyme was distilled over sodium and stored under nitrogen atmosphere over molecular sieves (4 Å). Styrene and divinylbenzene were degassed and stored under nitrogen. Prior to use both were purified through column chromatography over aluminium oxide to remove the inhibitor. All other chemicals were used as received.

### 2.2. Synthesis

#### 2.2.1. 4-Vinyl Benzyl Alcohol (VBA)

4-Vinylbenzyl chloride (31.62 g, 0.2072 mol) and potassium acetate (23.34 g, 0.2378 mol) were dissolved in DMSO (70.50 mL). Hydroquinone (11.9 mg, 1.081 m mol) was added as a stabilizer. The resulting solution was stirred for two days at 40 °C before it was filtered and the filtrate was extracted with chloroform. Subsequently, the organic layer was washed with demineralized water. The combined organic layers were dried over Na_2_SO_4_, filtrated and removed in vacuo. Afterwards the residue was dissolved in ethanol (94.50 mL) and H_2_O (14.61 g, 0.8108 mol), NaOH (14.63 g, 0.3658 mol) and hydroquinone (11.6 mg, 1.081 mmol) were added. The resulting solution was refluxed at 100 °C for 90 min before it was cooled down to room temperature. The solution was filtered and extracted with chloroform. The organic phase was washed three times with demineralized water, dried over Na_2_SO_4_ and filtrated. Chloroform was removed in vacuo and the residue was fractionally distilled at 110 °C under high vacuum to yield the product as a light-yellow solid. Yield: 42%. *δ_H_* (300 MHz, CDCl_3_) = 7.42 (d, 2H, Ar*H*), 7.33 (d, 2H, Ar*H*), 6.78–6.64 (dd, 1H, C*H*), 5.79–5.70 (d, 1H, C*H*_2_), 5.27–5.21 (d, 1H, C*H*_2_) and 4.65 ppm (s, 2H, C*H*_2_).

#### 2.2.2. VBA-PEEGE Macromonomer 1a

VBA (1.65 g, 12.3 mmol) was dissolved in diglyme (11 mL, 76.8 mmol) under nitrogen atmosphere before 1 M K^t^BuOH (2.45 mL, 2.45 mmol), dissolved in THF was added to the solution. The resulting solution was stirred for half an hour at room temperature before *tert*-butanol and THF were removed under vacuum. Thereafter, EEGE (10.8 g, 73.8 mmol) was added to the remaining solution. The resulting reaction mixture was stirred overnight at 80 °C before cooling to room temperature. The excess solvents were removed under vacuum yielding the product as an oil. VBA-PEEGE macromonomers **1b** and **1c** were synthesized using the same procedure (Table 1). Yield: 79%. *δ_H_* (300 MHz, CDCl_3_) = 7.39 (d, 2H, Ar*H*), 7.29 (d, 2H, Ar*H*), 6.75 (q, 1H, C*H*), 5.77 (d, 1H, C*H*_2_), 5.25 (d, 1H, C*H*_2_), 4.70 (t, 6H, C*H*), 4.52 (s, 2H, C*H*_2_), 3.65–3.45 (m, 42H, C*H*_2_, C*H*_2_, C*H*_2_, C*H)*, 1.29 (d, 18H, C*H*_3_) and 1.19 ppm (t, 18H, C*H*_3_).

#### 2.2.3. VBA-Oligoglycidol Macromonomer 2a

VBA-PEEGE_6_ **1a** (9.78 g, 9.7 mmol) was dissolved in THF (325 mL) before 37% HCl (16 mL, 0.52 mol) was added to the solution. The reaction mixture was stirred for three hours at room temperature before it was neutralized using saturated aq. NaHCO_3_. Thereafter, the mixture was filtered and the filtrate was evaporated under vacuum yielding the product as a viscous dark orange liquid. VBA-Oligoglycidol macromonomers **2b** and **2c** were synthesized using the same procedure starting from VBA-PEEGE **1b** and **c**, respectively. Yield: 99%. *δ_H_* (300 MHz, CDCl_3_) = 7.56 (d, 2H, Ar*H*), 7.43 (d, 2H, Ar*H*), 6.87 (q, 1H, C*H*), 5.92 (d, 1H, C*H*_2_), 5.37 (d, 1H, C*H*_2_), 4.60 (s, 2H, C*H*_2_) and 3.79–3.60 ppm (m, 30H, C*H*_2_, C*H*_2_, C*H*).

#### 2.2.4. Prototypical Synthesis of Polystyrene Nanoparticles

VBA-oligoglycidol was weighed under a nitrogen atmosphere. Degassed H_2_O was added (100 mL) and the solution was stirred until all VBA-oligoglicidol was dissolved. Subsequently, degassed styrene (5.0 g, 5.5 mL, 48 mmol) and DVB (0.27 mL, 1.9 mmol) were added to the reaction mixture. The reaction mixture was then stirred and heated to 80 °C before a solution of potassium persulfate (50 mg, 0.18 mmol) in degassed H_2_O (2 mL) was added. The reaction was stirred overnight at 80 °C. Additionally, after cooling down, the emulsion was filtered to remove large agglomerates. The filtrate was centrifuged and dispersed in water via ultrasonic treatment at room temperature for 1 min. All emulsion polymerizations with varying surfmer concentration and chain length were conducted as described with the combination of surfmer type and amount listed in Table 2.

### 2.3. Colloidal Stability Testing

For the colloidal stability tests polystyrene nanoparticles with oligoglycidol_6_ (**2a**) and an average particle size of 243 and 369 nm were synthesized for the comparison with nanoparticles **4b** and **5** bearing oligoglycidol_10_ (**2b**) and oligoglycidol_20_ (**2c**) and average particle size of 273 and 405 nm respectively.

First, nanoparticle dispersions of 0.5 wt % solid content were prepared for all samples to be tested. Then sodium chloride solutions with a concentration between 0.02 and 6 mol/L were prepared. Next 50:50 mixtures of the particle dispersions and sodium chloride solutions were prepared to get to the desired particle and sodium chloride concentrations. For the stability tests with a sodium chloride concentration of 5 mol/L, the solution with 6 mol/L sodium chloride was mixed with small amounts of highly concentrated particle dispersions to reach a particle content of 0.25 wt % and the desired sodium chloride concentration. After mixing particle dispersions and sodium chloride solutions, the average particle size was measured by dynamic light scattering (DLS). When large agglomerates (≥10× single particle diameter) were formed at a certain sodium chloride concentration, the stability test was stopped for this type of nanoparticle.

### 2.4. Characterization and Measurement Methods

#### 2.4.1. Nuclear Magnetic Resonance Spectroscopy (NMR)

^1^H NMR spectra were recorded on a Bruker DPX-400 FT-NMR spectrometer (Billerica, MA, USA) at 300 MHz. chloroform (CDCl_3_) was used as a solvent.

#### 2.4.2. Surface Tension Measurements

Surface tension was measured via pendant drop measurements using an Attension Theta Flex optical tensiometer from Biolin Scientific (Gothenburg, Sweden) with an accuracy of ±0.01 mN/m. The measurements were conducted at room temperature and solutions were prepared at least 2 h before measurement.

#### 2.4.3. Dynamic Light Scattering (DLS)

Particle sizes and polydispersity indices (PDIs) were measured using DLS. Measurements (Malvern Instruments, Malvern, UK) were performed at 25 °C using a Zetasizer Nano Series from Malvern Instruments with an accuracy of < ± 2% using a quartz glass cell for optical measurements (Malvern Instruments, Malvern, UK).

#### 2.4.4. Zeta Potential Measurements

Zeta potential were performed at 25 °C using a Zetasizer Nano Series from Malvern Instruments using a zeta potential cell (Malvern Instruments, Malvern, UK).

#### 2.4.5. Scanning Electron Microscopy (SEM)

SEM images were partly acquired using a Teneo SEM from Thermo Fisher Scientific (Thermo Fischer Scientific, Waltham, MA, USA). The measurements were conducted at Enabling Technologies. Other images were acquired using a Hitachi S4800 FESEM or a FEI quanta 600 microscope. For sample preparation one droplet of the particle dispersion was placed on a silicon wafer and dried at room temperature. The samples were sputtered with gold. Particle diameters and standard deviations from SEM images were determined using MATLAB. For average diameter and standard deviation at least 200 particles per sample were measured.

#### 2.4.6. Headspace Gas Chromatography–Mass Spectroscopy (HS-GC/MS)

Headspace gas chromatography–mass spectroscopy (HS-GC/MS) measurements were performed using a Perkin Elmer Clarus 500 gas chromatograph (Perkin Elmer, Waltham, MA, USA) with a Restek Rtx-VRX (60 M × 0.25 mm, 1.4 µm film) analytical column and a Perkin Elmer Clarus 500 Mass Spectrometer detector (Perkin Elmer, Waltham, MA, USA). The GC oven program was as follows: Initial 70 °C, hold 3 min. Ramp 10 °C/min to 230 °C, hold 5 min. For sample preparation part of 4.78 wt % polystyrene latex in water was weighed in duplicate and dissolved in 100 mL of water. A standard addition curve was made for identification and quantification of styrene (CAS 100–42–5) in the sample. 500 µL of both sample solutions were spiked multiple times with calibration standards and were conditioned for 20 min at 80 °C. After conditioning the spiked sample solutions were analysed with HS-GC/MS.

## 3. Results and Discussion

### 3.1. Surfmer Synthesis and Analysis

**Figure 1:**
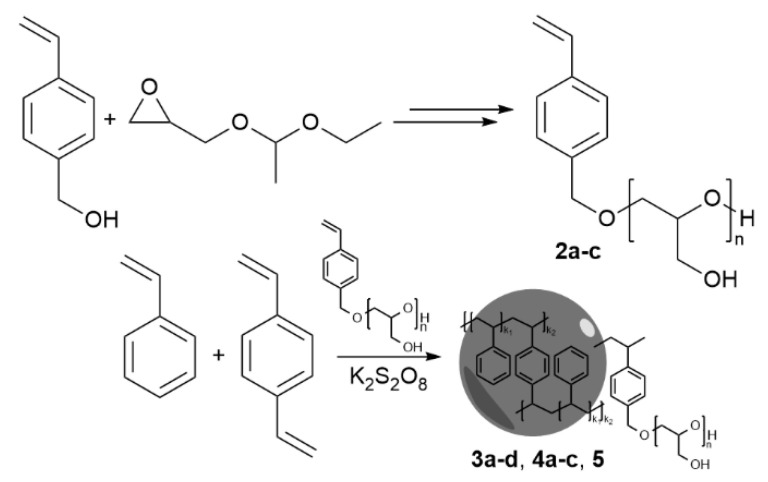
Reaction scheme of oligoglycidol-functionalised styrene surfmer synthesis, and schematic representation of subsequent emulsion polymerisation of styrene and divinyl benzene (*n* = 6, 10 and 20, for **2a**–**c** respectively).

To study the influence of the oligoglycidol chain length on the surfactant properties of the oligoglycidol-functionalised styrene macromolecule, we synthesized surfmers with three different oligoglycidol chain lengths according to the previously reported procedure [27]. We used vinyl benzyl alcohol (VBA, see Figure S1) and ethoxy ethyl glycidyl ether (EEGE) in ring opening polymerisation to yield poly(ethoxy ethyl glycidyl ether) (PEEGE) **1a**–**c** with 6, 10 and 20 repeating units (Figure 1). The polymer chain length was controlled by adjusting the VBA:PEEGE ratio, and it was analysed by ^1^H-NMR (see Figure S2). The oligoglycidol-functionalised surfmers **2a**–**c** were obtained via acidic deprotection of the PEEGE molecules. After removing the acetal protecting groups the oligoglycidol chain length was again determined via ^1^H-NMR. For surfmers **2a**–**c** NMR analysis resulted in oligoglycidol chain lengths of 6, 10 and 20 repeating units, respectively (see Figure S3). Furthermore, we performed mass spectroscopy (MS) to analyse the polydispersity of the synthesized macromonomers of oligoglycidol **2a**–**c** (see Figures S4–S6). The MS analysis revealed that for all macromonomers not only one chain length was obtained but a distribution of chain lengths, and that the number of repeating units calculated from NMR are average values.

**Figure 2:**
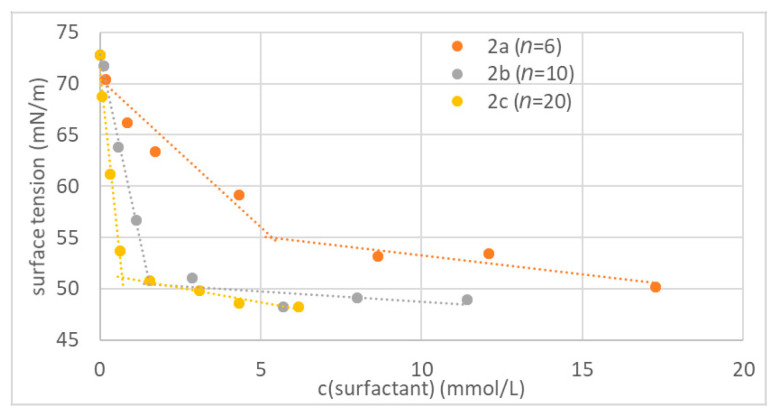
Surface tension as function of the surfmer concentration in water (*n* = number of repeating units in the oligoglycidol moiety of the surfmer; intersection of regression lines at critical micelle concentration (CMC)).

To study the surfactant properties of the three synthesized oligoglycidol surfmers **2a**–**c** with 6, 10 and 20 repeating units, we performed pendant drop measurements on aqueous solutions at various concentrations of each macromolecule. Here it was important to prepare the solutions 2 h before measurement to reach stable surface tension values. For all three surfactants the surface tension decreased rapidly with increasing concentration before it reached values between 50 and 55 mN·m^−1^ at the CMC (Figure 2), where further increase in surfactant concentration only led to a small decrease in surface tension. With increasing oligoglycidol chain length the CMC was reached at a lower concentration. Therefore, the surfactant properties of oligoglycidol surfmer increased with increasing chain length. For the macromolecules **2a**–**c** with 6, 10 and 20 repeating units CMCs of 5.38, 1.55 and 0.69 mmol·L^−1^, respectively, were determined. The results obtained via surface tension measurements suggest that an increase in oligoglycidol chain length leads to an increase in hydrophobicity of the macromolecule, since according to Traube’s rule an increase in hydrophobicity of the surfactant will lead to an increase in surface activity [30]. An increase in oligoglycidol chain length increased both the number of polar OH groups and the number of apolar CH_2_ moieties in the surfmer. The results obtained via surface tension measurements suggest that the influence of the apolar moieties dominates, and therefore the overall hydrophobicity of the surfactant increased with increasing oligoglycidol chain length. The surfactant **2a** with the CMC at the highest concentration was used to study the influence of oligoglycidol surfmer concentration on the particle size below the CMC.

### 3.2. Polystyrene Nanoparticle Synthesis

#### 3.2.1. Influence of Surfmer Concentration Below the CMC on Nanoparticle Size

**Figure 3:**
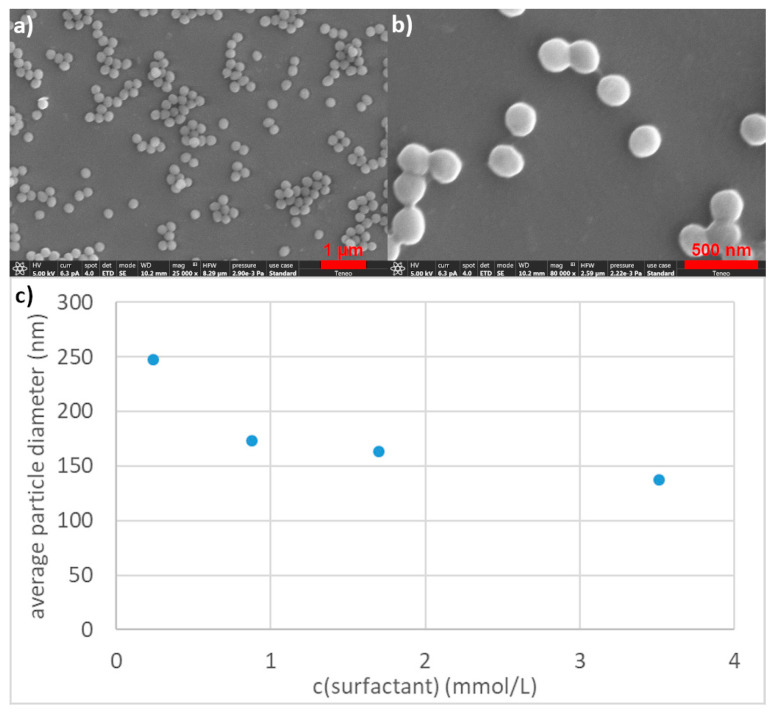
(**a**) Overview and (**b**) high resolution SEM image of nanoparticles **3a** synthesized with 0.24 mmol·L^−1^ of oligoglycidol_6_ (**2a**) as surfmer, and (**c**) average particle size (determined by dynamic light scattering (DLS)) as a function of the surfmer concentration (surfmer **2a** with 6 repeating units in all experiments).

For the surfmer **2a**, we selected four different surfmer concentrations below the CMC, and synthesized polystyrene nanoparticles **3a**–**d** in an emulsion polymerisation (Figure 1 and Figure 3a,b). We used potassium persulfate as a radical initiator and added small amounts of divinyl benzene to enable cross linking. The emulsion polymerizations were performed for 24 h at 80 °C for maximum conversion of the monomers. At this temperature the surfmer **2a** was still completely soluble and the cloud point wasn’t reached. The degree of conversion was analysed by headspace GC–MS on the final latex. Here 0.73 wt % monomer were detected (see Figure S7), which is in the range of commercial polystyrene latexes [31]. All starting material concentrations and initial conditions were kept constant for these reactions with different surfmer concentrations (Table 2).

Emulsion polymerisation with **2a** at a concentration of 0.24 mmol·L^−1^ and a surfmer:monomer ratio of 1:1984 resulted in spherical particles **3a** with an average diameter of 247 nm and a narrow size distribution with a PDI of 0.015 (Figure 3a,b and Table 3). The particle size was analysed by dynamic light scattering (DLS) and was confirmed by scanning electron microscopy (SEM, Table 3). The average particle diameter obtained from SEM images was with 235 nm slightly smaller than the average diameter obtained via DLS (Table 3). A zeta potential of −37.7 mV was measured for polystyrene particles **3a**, which illustrates that surface bound sulphate groups of the initiator offer additional charge stabilisation. Increasing the surfmer concentration to 0.88 mmol·L^−1^ and the surfmer:monomer ratio to 1:545 leads to a reduction in particle size and an average nanoparticle diameter of 173 nm with a PDI of 0.004 (as determined by DLS; **3b**, Figure S8, Table 3). A further increase in surfmer concentration yielded nanoparticles **3c** and **d** with even smaller diameters of 163 and 137 nm (determined by DLS, Figures S9 and S10, Table 3) respectively. Both nanoparticles **3c** and **d** showed a narrow size distribution with PDI of 0.009 and 0.010 respectively (Table 3). Hence the overall trend showed that an increase in surfmer concentration led to a reduction in particle size if all other reaction parameters are kept constant (Figure 3c). As previously reported [16], below the CMC homogeneous nucleation of surfmer molecules reacting with the initiator drives the polymerisation. Therefore an increase in surfmer concentration led to a higher nucleation rate and therefore to smaller particles. All synthesized nanoparticles showed a narrow size distribution in DLS as well as SEM analysis and similar zeta potential (Table 3).

#### 3.2.2. Influence of A Change in the Nucleation Mechanism on Nanoparticle Size

**Figure 4:**
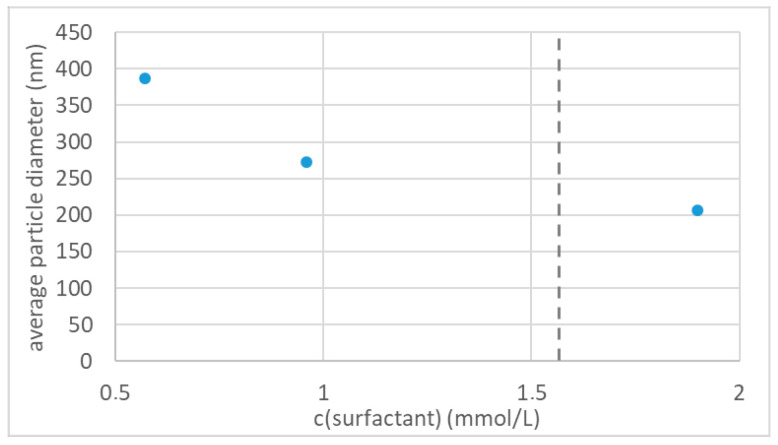
Average particle size (determined by DLS) dependant on surfactant concentration for oligoglycidol_10_ (**2b**). At a concentration of 1.55 mmol·L^−1^ (dashed line) the CMC is reached.

Furthermore, we applied the oligoglycidol macromolecule **2b** with a CMC at 1.55 mmol·L^−1^ to study the influence of the different reaction mechanisms above and below the CMC on the particle size. We chose two surfmer concentrations below and one concentration above the CMC in emulsion polymerisations with the same starting material concentrations and reaction conditions as mentioned before. Additionally, for surfmer **2b** the cloud point was not reached at 80 °C and the surfmer was completely soluble in water. The synthesis with 0.57 mmol·L^−1^ oligoglycidol_10_ surfmer **2b** and a surfmer:monomer ratio of 1:840 resulted in spherical nanoparticles **4a** with an average diameter of 387 nm and a narrow size distribution with a PDI of 0.012 (as determined by DLS, Figure S11, Table 3). Increasing the surfmer concentration to 0.96 mmol·L^−1^ led, as for surfmer **2a**, to a reduction in average particle size. At this surfmer concentration nanoparticles **4b** with an average diameter of 273 nm and a PDI of 0.014 were obtained (as determined by DLS, Figure S12, Table 3). When the oligoglycidol_10_ surfmer concentration was increased further to pass the CMC the overall trend of reducing particle size continued (Figure 4). Here at a surfmer concentration of 1.9 mmol·L^−1^ particles **4c** with an average diameter of 206 nm and a PDI of 0.018 were obtained (as determined by DLS, Figure S13, Table 3). A doubling in the surfmer concentration led to a reduction in particle size by 25% when the CMC was passed. This is a much larger size reduction than for the oligoglycidol_6_ surfmer **2a** in the same concentration range, for which the CMC was not reached. In that case a doubling of surfmer concentration from 0.88 to 1.7 mmol·L^−1^ only led to a particle size reduction of 6% (Figure 3). Four times the amount of oligoglycidol_6_ surfmer **2a** was needed to reduce the average particle size by 21%. The large size reduction for oligoglycidol_10_ (**2b**) at 1.9 mmol·L^−1^ was therefore likely due to the change in the nucleation mechanism. By passing the CMC a large number of micelles were formed, which enable the initiator to start the polymerisation not only with surfmer molecules in solution but also with monomers in the micelles, which increased the nucleation rate drastically. Here, the emulsion polymerisation progresses via Smith–Ewart kinetics [28] with a high nucleation rate inside the micelles, and therefore particles with a much smaller average diameter are obtained. For all emulsion polymerizations above and below the CMC nanoparticles with a narrow size distribution were obtained (Table 3), and therefore no influence of nucleation mechanism on the polydispersity was observed.

#### 3.2.3. Influence of Oligoglycidol Chain Length on Nanoparticle Size

**Figure 5:**
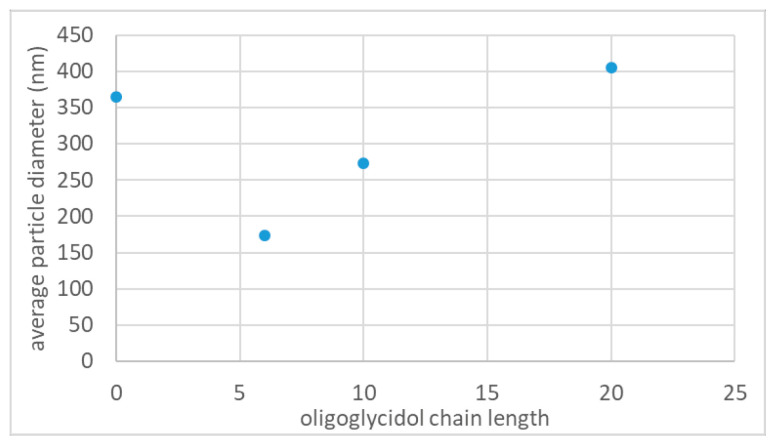
Average polystyrene particle size (determined by DLS) as a function of the oligoglycidol chain length at surfmer concentrations around 1 mmol·L^−1^ and surfmer:monomer ratios around 1:500 (datapoint at *n* = 0 represents control experiment without a surfmer).

Additionally, we analysed the influence of the oligoglycidol chain length on the average polystyrene particle size whilst keeping the surfmer concentration constant. Therefore, we synthesized, in addition to the previously described particles, polystyrene nanoparticles **5** with 0.96 mmol·L^−1^ oligoglycidol_20_ (**2c**). Keeping all other reaction parameters constant led to nanoparticles with an average particle diameter of 405 nm and a narrow size distribution with a PDI of 0.037 (as determined by DLS, Figure S14, Table 3). As for the other surfmers, **2c** was still completely soluble in water at 80 °C. Furthermore we performed a control experiment using previously described reaction parameters without the addition of surfmer. Here, polystyrene nanoparticles **6** with an average diameter of 365 nm and a PDI of 0.18 (as determined by DLS, Figure S15, Table 3) were obtained. The nanoparticles **6**, synthesized without surfactant, showed a very large size distribution. In SEM analysis particles with diameters ranging from approximately 50 to 500 nm were observed (see Figure S15). With this experiment we could show that latex particles can be stabilized solely by the sulphate group of the used initiator, but that the stabilization is not enough to get good control over the particle size. Therefore a large range of differently sized particles was obtained. Comparing nanoparticles of all three oligoglycidol surfmers **2a**–**c** with a concentration of approximately 1 mmol·L^−1^ and a surfmer:monomer ratio around 1:500, a clear trend was visible (Figure 5). It can be observed that an increase in the chain length led to an increase in the average particle size (Figure 5). Furthermore, comparing the average particle size of latexes synthesized with and without surfmer it can be seen that the use of **2c** as surfmer led to nanoparticles with a similar average diameter as using no surfmer. Together with the surface tension measurements, which suggest that an increase in the oligoglycidol chain length leads to an increase in hydrophobicity, it can be proposed that with increased chain length the oligoglycidol macromolecules have an increased tendency to be located inside the polystyrene nanoparticles instead of at the surface. This tendency, caused by increased hydrophobicity, would lead to less amount of surfactant being available to stabilize the nanoparticles and therefore larger particles. Another factor, which may influence the resulting particle size, is the reactivity of the oligoglycidol surfmer. As described in a previous article [16], the reaction mechanism of emulsion polymerisation of styrene with oligoglycidol as a surfmer goes via a homogeneous nucleation mechanism, when the surfmer concentration is below the CMC. Here, in the initial stage of the polymerisation the rate of nucleation is determined by the reactivity of the oligoglycidol-functionalised surfmer. A change in oligoglycidol chain length can influence the reactivity due to, e.g., larger steric hindrance of the vinyl group. Furthermore surfmer **2c** is at a concentration of 1 mmol·L^−1^ already above the CMC, whereas surfmers **2a** and **b** are still below. Therefore the difference in reaction mechanism will also have an influence on the different particle sizes. Nevertheless additional experiments investigating the hydrophobicity of the different oligoglycidol surfmers and the nucleation rate of emulsion polymerisations using those surfmers at various concentrations are necessary for a better understanding.

### 3.3. Colloidal Stablity of Various Latex Dispersions

#### 3.3.1. Influence of Particle Size on Colloidal Stability

**Figure 6:**
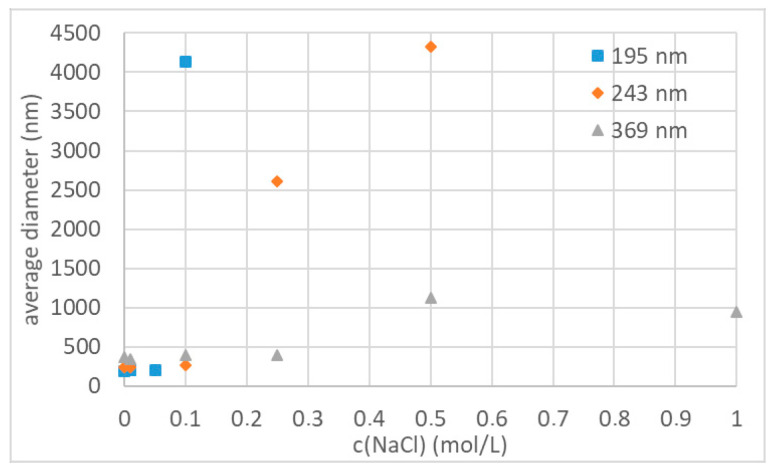
Colloidal stability of different sized nanoparticles with oligoglycidol_6_ (**2a**) surface functionality in various NaCl concentrations.

After synthesizing polystyrene nanoparticles of various sizes and stabilized with different oligoglycidol surfmers, we analysed the colloidal stability of the nanoparticle dispersions in aqueous NaCl solutions. Therefore, we used nanoparticle dispersions at a distinct solid content and added those to NaCl solutions of several concentrations. Afterwards we immediately conducted DLS measurements to analyse the average particle size, which was recorded 3 min after sample preparation. Firstly, we analysed the colloidal stability as a function of the particle size. For this, we used three particle dispersions functionalised with **2a** and an average particle size between 195 and 369 nm (**3e**–**g**, respectively). We analysed the colloidal stability of each of these particle dispersions at NaCl concentrations between 0.01 and 5 mol·L^−1^. The particles with the smallest particle size of 195 nm were stable up to a NaCl concentration of 0.05 mol·L^−1^ (Figure 6, blue squares). Upon a further increase in NaCl concentration the particles immediately started to form large agglomerates. Larger particles, which are prepared with less surfmer, displayed an improved colloidal stability (Figure 6, orange diamonds and grey triangles). The polystyrene particles with an average size of 243 nm (Figure 6, orange diamonds) were still stable at an NaCl concentration of 0.1 mol·L^−1^ and first started to form medium sized agglomerates at an NaCl concentration of 0.25 mol·L^−1^ before large agglomerates were formed at 0.5 mol·L^−1^. This trend continued further for increasing particle size. The nanoparticles with an average diameter of 369 nm (Figure 6, grey triangles) were still stable at 0.25 mol·L^−1^ NaCl, only started to form clusters of 2–3 particles at a concentration of 0.5 mol·L^−1^ and further increase of NaCl concentration did not impact the colloidal stability further. Up to a NaCl concentration of 5 mol·L^−1^ only clustering of 2–3 particles occurred without formation of large agglomerates.

#### 3.3.2. Time Dependency of Colloidal Stability

**Figure 7:**
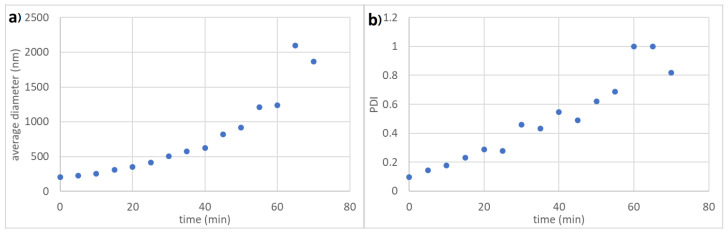
(**a**) Average particle size and (**b**) PDI over time of nanoparticles with 195 nm average diameter and oligoglycidol_6_ surface functionality at 0.05 mol·L^−1^ NaCl.

In addition to the observation that an increased particle size leads to increased colloidal stability we also observed a time dependency of the dispersions stability. To analyse this we measured the average particle size and the PDI of the latex particle dispersion with an average diameter of 195 nm and oligoglycidol_6_ (**2a**) surface functionality at 0.05 mol·L^−1^ NaCl over time. Here we observed that for the first 10 min the average particle size stayed constant before it slowly started to increase (Figure 7a). Up to 25 min the average particle size indicates only dimers and agglomerates of more than 5 particles only start to appear after 55 min. Even though the average particle size suggest only a slow agglomeration, the development of the PDI over time demonstrates that the agglomeration starts immediately because a steady increase in PDI can be observed (Figure 7b). Due to the time dependency of colloidal stability of latex dispersions it is important to measure average particle sizes at the same time after sample preparation if different concentrations and particles should be compared. Therefore, we chose to record all average particle sizes in colloidal stability experiments 3 min after sample preparation.

#### 3.3.3. Influence of Oligoglycidol Chain Length on Colloidal Stability

**Figure 8:**
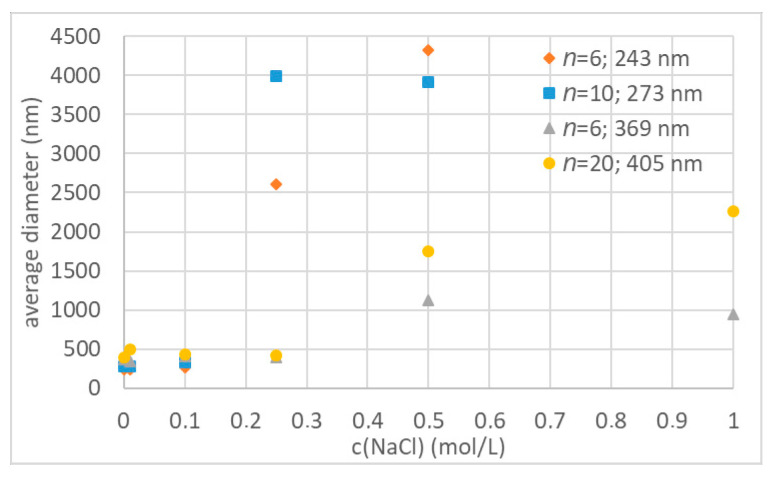
Colloidal stability for nanoparticles with different surface functionality.

Furthermore, we analysed the influence of the oligoglycidol chain length on the colloidal stability of polystyrene nanoparticles. Therefore, we used the particle dispersions (**4b**, **5**) with an average diameter of 273 and 405 nm and surface functionalised with surfmers **2b** and **2c** respectively (Figure 8, blue squares and yellow dots, respectively), in the above described experiment and compared the results to the stability test of **2a** functionalised particles with an average diameter of 243 nm and 369 nm respectively (Figure 8, orange diamonds and grey triangles, respectively). Both particle dispersions functionalised with **2b** and **2c** showed the same colloidal stability as particles functionalised with **2a** and similar particle size (Figure 8). The nanoparticles synthesized with **2b** and an average diameter of 243 nm were stable up to a NaCl concentration of 0.1 mol·L^−1^ and started to agglomerate at 0.25 mol·L^−1^. The nanoparticles synthesized with **2c** and an average diameter of 405 nm were colloidally stable up to an NaCl concentration of 0.5 mol·L^−1^ and only formed clusters of 2–3 particles for higher concentrations up to 5 mol·L^−1^. Ergo, no effect of oligoglycidol chain length on the colloidal stability was observed.

The colloidal stability of all oligoglycidol functionalised polystyrene nanoparticles is based on a combination of surface charge due to the sulphate groups originating from the initiator, and the oligoglycidol surfmer. The charge stabilisation is negated already when small amounts of ions are present in the solution, which we could show by investigating the colloidal stability of nanoparticles **6** at low NaCl concentrations. These particles, synthesized without the use of surfactant, already started to agglomerate at NaCl concentrations of 0.01 mol·L^−1^. Regarding the influence of different oligoglycidols on the colloidal stability, we initially expected an increase in the oligoglycidol chain length to lead to an increased hydrophilicity and therefore smaller, and colloidally more stable polystyrene particles. The results obtained however show that, contrary to our expectation, an increase in oligoglycidol chain length leads to increased hydrophobicity of the surfmer and to larger particles when used in an emulsion polymerisation with styrene. A significant effect of oligoglycidol chain length on the colloidal stability of functionalized styrene particles, when subjected to NaCl solutions, could not be observed. For the same oligoglycidol chain length, our results show that an increase in surfmer concentration leads to decreasing particle size. Furthermore the particles with the largest diameter, which are obtained with the least amount of surfactant, show the highest colloidal stability in the presence of NaCl.

## 4. Conclusions

We conducted a systematic study to analyse the impact of the concentration and chain length of oligoglycidol-based surfmers on the emulsion polymerisation of styrene. We studied the influence of surfmer concentration on the particle size, both for polymerisations carried out at concentrations below and above the critical micelle concentration (CMC). Additionally, we analysed the influence of oligoglycidol chain length on the average particle size whilst keeping the surfmer concentration constant. Our study unveiled that below the CMC the average particle size decreased with increasing surfmer concentration. Here, an increase in surfmer molecules led to a higher homogeneous nucleation rate. When the CMC passed, a more steep decrease in particle size occurred due to a change in nucleation mechanism. Being above the CMC heterogeneous nucleation inside the formed micelles—according to the mechanism proposed by Smith and Ewart—led to a large increase in the nucleation rate, and therefore a large decrease in particle size. Furthermore, an increase in particle size with increasing oligoglycidol chain length was observed whilst keeping the concentration of the surfmer constant. This can be explained by increased hydrophobicity of oligoglycidol with increased chain length, and therefore an increased tendency to be buried inside the polystyrene nanoparticle instead of being positioned at the particle–water interface. Furthermore, an influence of the oligoglycidol chain length on the reactivity of the surfmer can play a role in the rate of nucleation. Additionally, we analysed the colloidal stability of polystyrene nanoparticles in aqueous NaCl solutions as function of particle size and oligoglycidol chain length. Our study showed that an increase in particle size led to an increase in colloidal stability. At average particle sizes around 350 nm no large agglomerates were formed even at NaCl concentrations of 5 mol·L^−1^. An effect of the oligoglycidol chain length on colloidal stability could not be observed. In general it can be concluded that colloidal stability of all oligoglycidol functionalised polystyrene nanoparticles is based on a combination of surface charge due to sulphate groups originating from the initiator, and the oligoglycidol surfmer. The charge stabilisation did not contribute to the colloidal stability in NaCl, whereas the addition of oligoglycidol led to increased colloidal stability. Contrary to our expectation, an increase in oligoglycidol chain length led to increased hydrophobicity of the surfmer and to larger particles. For the same oligoglycidol chain length an increase in surfmer concentration led to decreasing particle size, and the largest particles, synthesized with the least amount of surfactant, show the highest colloidal stability.

## Figures and Tables

**Table 1:** Synthesis of VBA-PEEGE macromonomers at 80 °C.

	VBA (g) (mmol)	Diglyme (mL)	K^t^BuOH ^a^ (mL) (mmol)	EEGE (g) (mmol)
**1a**	1.65 (12.3)	11	2.45 (2.45)	10.8 (73.8)
**1b**	1.65 (12.3)	11	2.45 (2.45)	18.0 (123)
**1c**	1.65 (12.3)	11	2.45 (2.45)	35.9 (246)

^a^ 1 M solution in THF.

**Table 2:** Synthesis of polystyrene nanoparticles.

	Oligoglycidol (*n* =) ^a^	Weight (mg)	Amount of Substance (mmol)	Concentration (mmol/L)	Surfmer:Monomer Ratio
**3a**	**2a** (6)	14	0.024	0.24	1:1984
**3b**	**2a** (6)	51	0.088	0.88	1:545
**3c**	**2a** (6)	98	0.17	1.7	1:283
**3d**	**2a** (6)	203	0.35	3.5	1:137
**3e**	**2a** (6)	33	0.057	0.57	1:842
**3f**	**2a** (6)	14	0.024	0.24	1:1984
**3g ^b^**	**2a** (6)	14	0.024	0.12	1:3968
**4a**	**2b** (10)	50	0.057	0.57	1:840
**4b**	**2b** (10)	84	0.096	0.96	1:500
**4c**	**2b** (10)	168	0.19	1.9	1:250
**5**	**2c** (20)	155	0.096	0.96	1:500

^a^*n* represents number of oligoglycidol units in the surfmer determined by ^1^H-NMR. ^b^ Particles **3g** were synthesized with doubling the volume of solvent and reactants.

**Table 3:** Synthesized polystyrene nanoparticles.

	Oligoglycidol (n =)	Surfmer: Monomer ratio	Average Diameter (DLS) (nm)	PDI ^a^ (DLS)	Average Diameter ^b^ (SEM) (nm)	Size Distribution ^c^ (SEM)	Zeta Potential (mV)
**3a**	**2a** (6)	1:1984	247	0.015	235	0.029	−37.7
**3b**	**2a** (6)	1:545	173	0.004	160	0.027	−46.9
**3c**	**2a** (6)	1:283	163	0.009	151	0.031	−44.6
**3d**	**2a** (6)	1:137	137	0.010	126	0.033	−45.9
**3e**	**2a** (6)	1:842	195	0.009			
**3f**	**2a** (6)	1:1984	243	0.012			
**3g**	**2a** (6)	1:3968	369	0.031			
**4a**	**2b** (10)	1:840	387	0.012	369	0.024	−42.3
**4b**	**2b** (10)	1:500	273	0.014	261	0.021	−46.5
**4c**	**2b** (10)	1:250	206	0.018	199	0.033	−44.3
**5**	**2c** (20)	1:500	405	0.037	389	0.041	−45.5
**6**	-	-	365	0.18	352	0.22	−46.3

^a^ The polydispersity index (PDI) describes the size distribution calculated from DLS measurements. ^b^ Average diameter calculated from SEM images using at least 200 particles. ^c^ Size distribution calculated from SEM images by dividing the standard deviation by average size of at least 200 particles.

## Supplementary Materials

The following are available online at https://www.mdpi.com/2073-4360/12/7/1557/s1, Figure S1–S3: 1H-NMR spectra of VBA, 1a and 2a. Figure S4–S6: LC-MS spectra of 2a–c. Figure S7: GC–MS chromatogram of latex 3a. Figure S8–S15: SEM images of particles **3b–d**, **4a–c**, **5** and **6**.
