# Supplementary material for: Oligoglycidol-Functionalised Styrene Macromolecules as Reactive Surfactants in the Emulsion Polymerisation of Styrene: The Impact of Chain Length and Concentration on Particle Size and Colloidal Stability
Source: Polymers (Basel). 2020 Jul 14;12(7):1557. doi: 10.3390/polym12071557 (PMC7408138; doi:10.3390/polym12071557)
Supplement: Supplementary file 1 [file polymers-12-01557-s001.pdf]

# Oligoglycidol-Functionalised Styrene Macromolecules as Reactive Surfactants in the Emulsion Polymerisation of Styrene: The Impact of Chain Length and Concentration on Particle Size and Colloidal Stability

Kim Waulthers,[1,‡] Ryan van Zandvoort,[2,3,‡] Sam Castermans,[1] Jeroen Welzen,[1] Evelien Baeten,[1] Kathleen Stout,[1] Helmut Keul,[4] Daniel Mann[2,3,*] and Pascal Buskens[1,2,3,5,*]

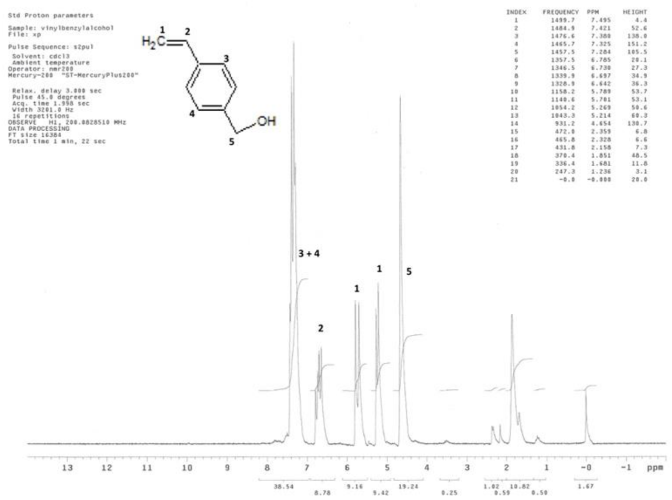


**Fig. S1** $^{1}$H-NMR of vinyl benzyl alcohol.

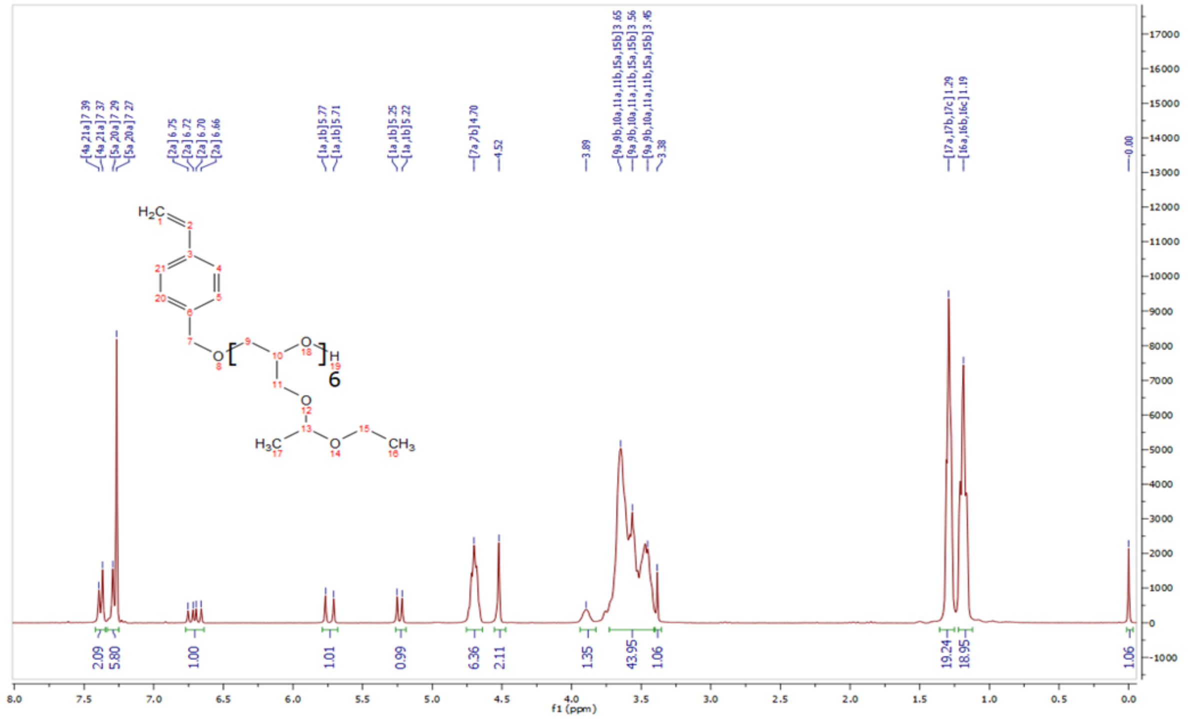


**Fig. S2** $^{1}$H-NMR of VBA-EEGE **1a** with average chain length of 6 EEGE repeating units. Average number of repeating units calculated from ratio between CH signal 2a at 6.71 ppm and signal around 3.56 ppm, representing $CH_2$ (9a,b), CH (10), $CH_2$ (11a,b) and $CH_2$ (15a,b).

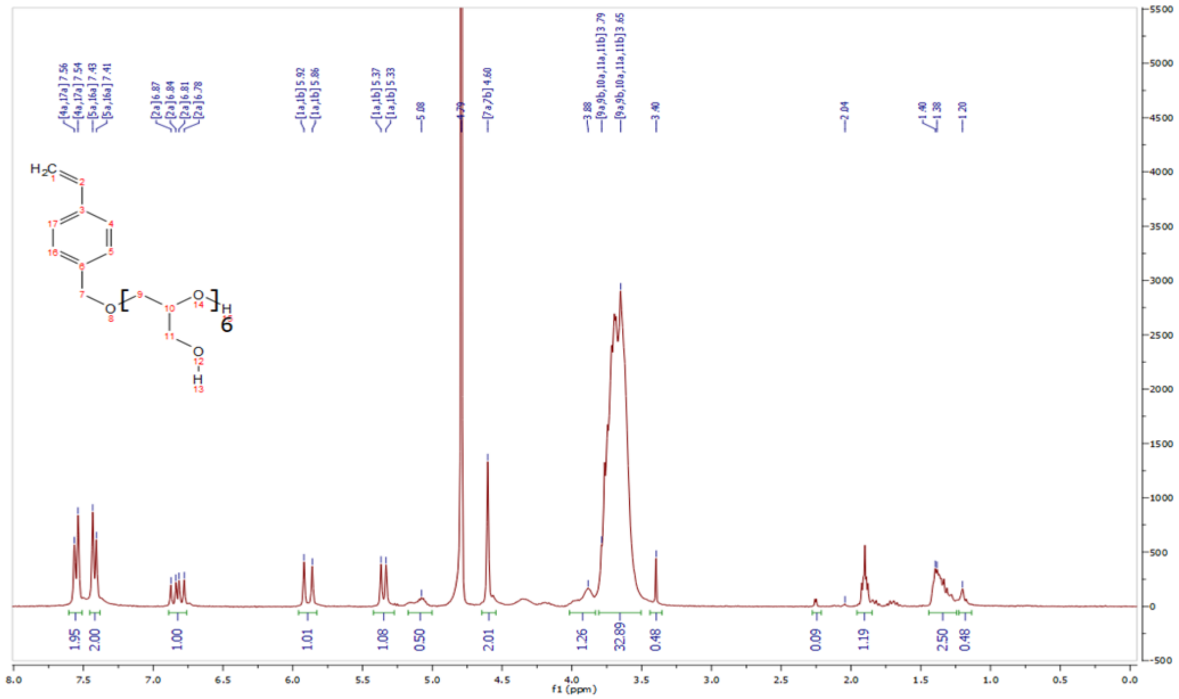

**Fig. S3** ¹H-NMR of VBA-oligoglycidol **2a** with average chain length of 6 EEGE repeating units. Average number of repeating units calculated from ratio between CH signal 2a at 6.82 ppm and signal around 3.70 ppm, representing $CH_2$ (9a,b), CH (10) and $CH_2$ (11a,b).

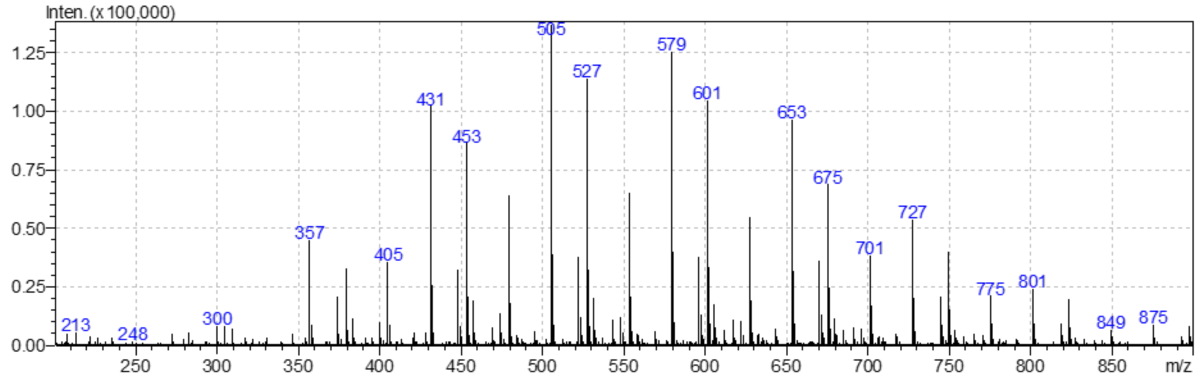

**Fig. S4** LC-MS spectrum of VBA-oligoglycidol **2a**.

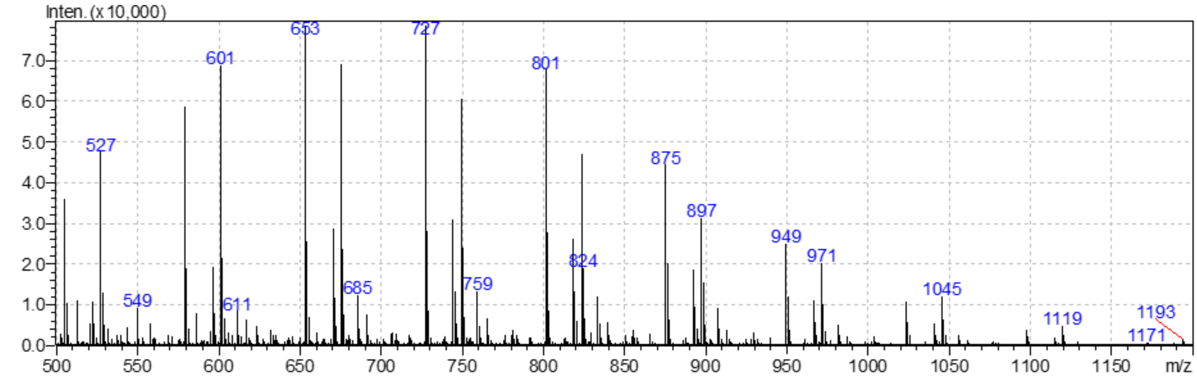

**Fig. S5** LC-MS spectrum of VBA-oligoglycidol **2b**.

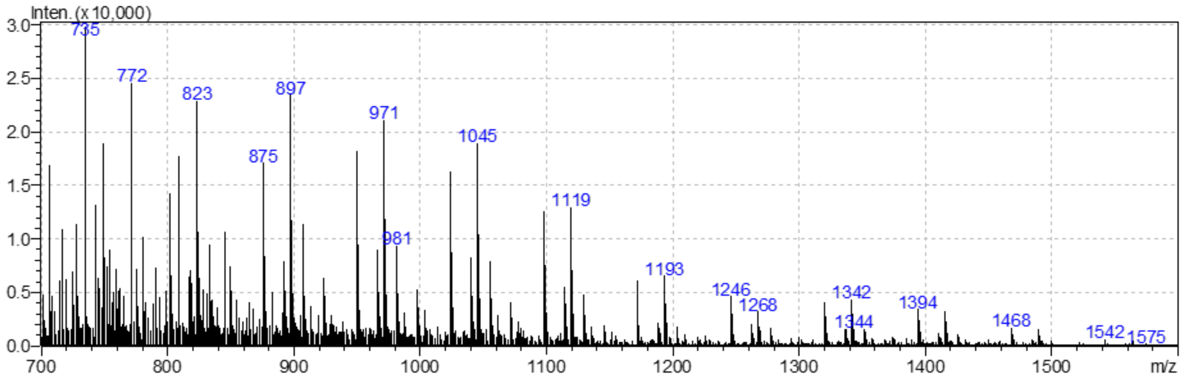


**Fig. S6** LC-MS spectrum of VBA-oligoglycidol **2c**.

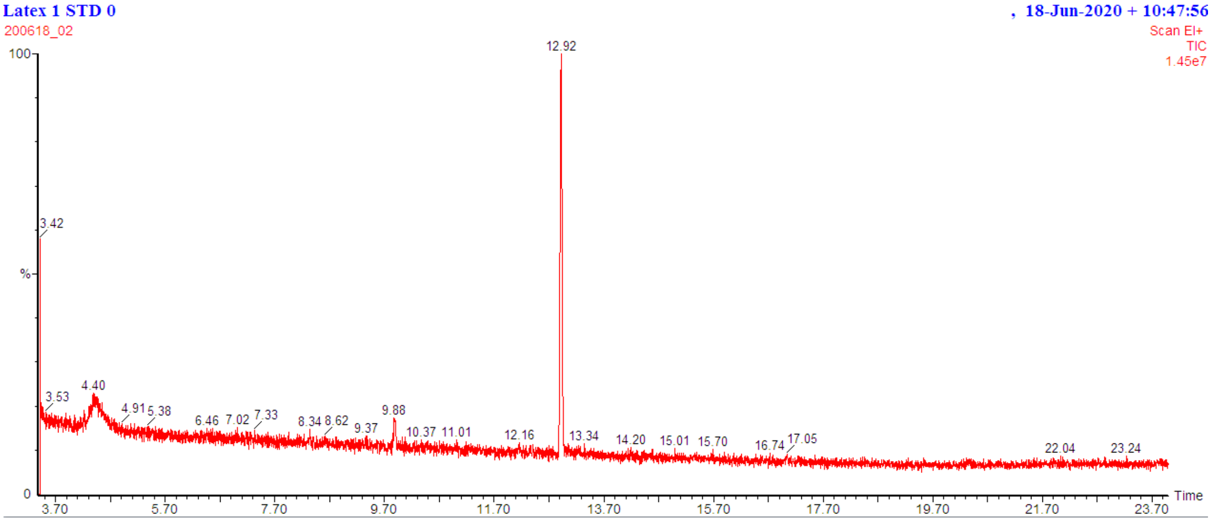


**Fig. S7** Headspace-GC-MS chromatogram of polystyrene nanoparticles **3a**.

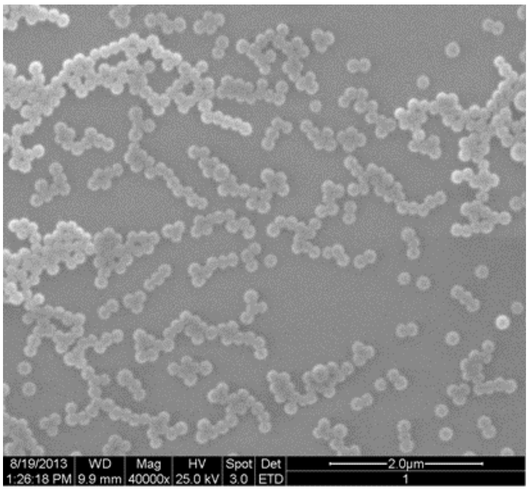


**Fig. S8** SEM image of nanoparticles **3b** synthesized with 0.88 mmol/L oligoglycidol$_6$ (**2a**).

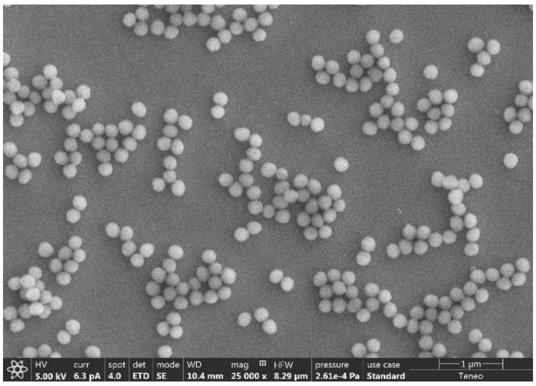

**Fig. S9** SEM image of nanoparticles **3c** synthesized with 1.7 mmol/L oligoglycidol$_6$ (**2a**).

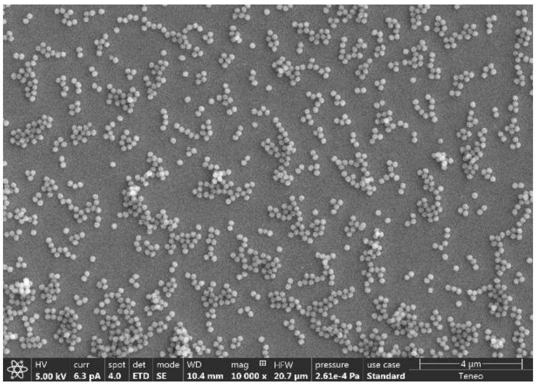

**Fig. S10** SEM image of nanoparticles **3d** synthesized with 3.5 mmol/L oligoglycidol$_6$ (**2a**).

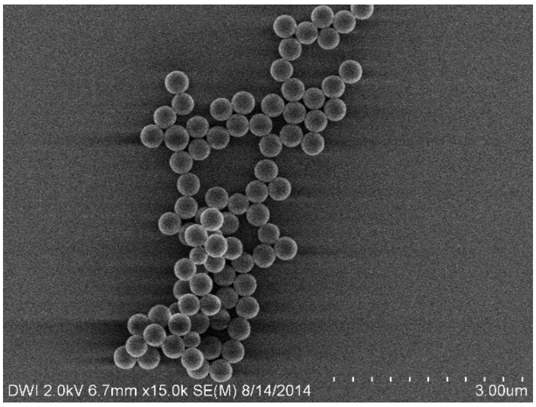


**Fig. S11** SEM image of nanoparticles **4a** synthesized with 0.57 mmol/L oligoglycidol$_{10}$ (**2b**).

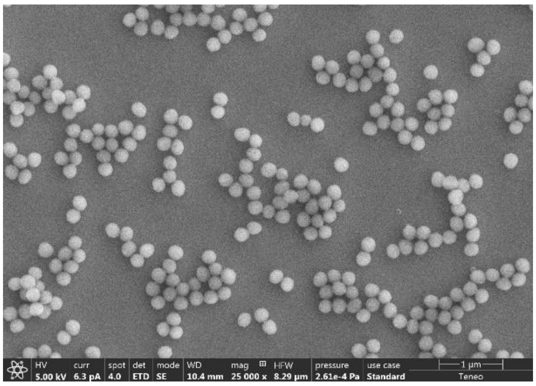

**Fig. S12** SEM image of nanoparticles **4b** synthesized with 0.96 mmol/L oligoglycidol$_{10}$ (**2b**).

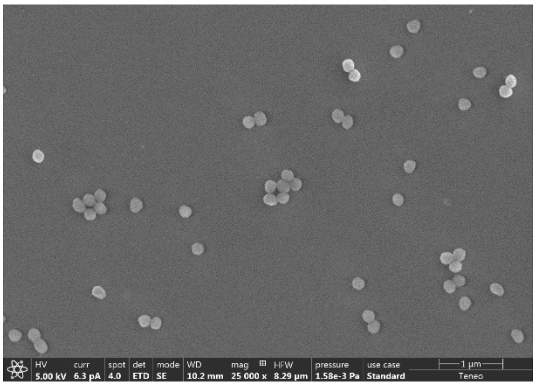


**Fig. S13** SEM image of nanoparticles **4c** synthesized with 1.9 mmol/L oligoglycidol$_{10}$ (**2b**).

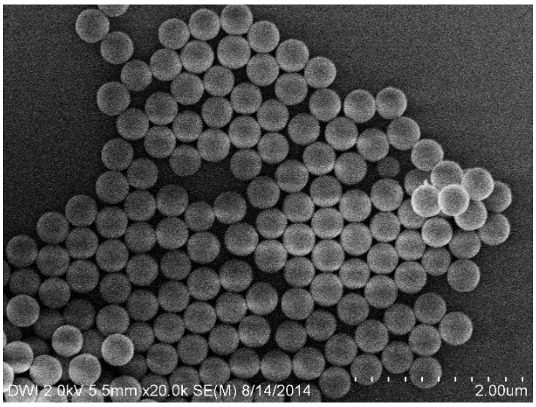


**Fig. S14** SEM image of nanoparticles **5** synthesized with 0.96 mmol/L oligoglycidol$_{20}$ (**2c**).

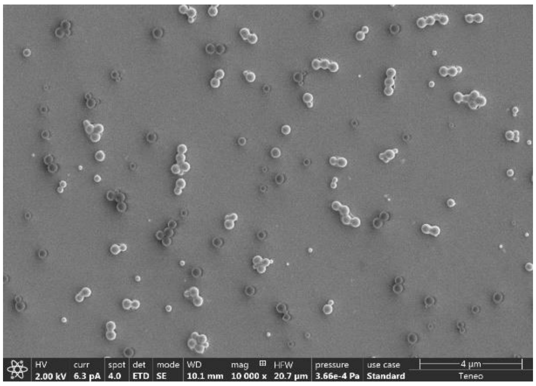


**Fig. S15** SEM image of nanoparticles **6** synthesized without addition of surfactant.
